# Spatial statistical analysis of regional disparities in suicide among policy units in Japan: Using the Bayesian hierarchical model

**DOI:** 10.1371/journal.pgph.0000271

**Published:** 2022-08-15

**Authors:** Masahide Koda, Katsunori Kondo, Satoru Takahashi, Toshiyuki Ojima, Tomohiro Shinozaki, Manabu Ichikawa, Nahoko Harada, Yasushi Ishida

**Affiliations:** 1 Division of Health Sciences, Center for Health Sciences and Counseling, Kyushu University, Fukuoka, Japan; 2 Center for Gerontology and Social Science, National Center for Geriatrics and Gerontology, Aichi, Japan; 3 Center for Preventive Medical Sciences, Chiba University, Chiba, Japan; 4 Department of Community Health and Preventive Medicine, Hamamatsu University School of Medicine, Shizuoka, Japan; 5 Department of Information and Computer Technology, Faculty of Engineering, Tokyo University of Science, Tokyo, Japan; 6 College of Systems Engineering and Science, Shibaura Institute of Technology, Tokyo, Japan; 7 Faculty of Interdisciplinary Science and Engineering in Health Systems, School of Nursing, Faculty of Health Sciences, Okayama University, Okayama, Japan; 8 Department of Psychiatry, Faculty of Medicine, University of Miyazaki, Miyazaki, Japan; PLOS: Public Library of Science, UNITED STATES

## Abstract

Suicide prevention is a crucial policy issue in Japan to be addressed nationally. Nevertheless, if there are regional differences in suicide, even in adjacent sub-regions, measures may need to be taken at the sub-regional level. Previous studies have not compared regional differences in suicide based on the size of policy units, such as prefectures, secondary medical areas, and municipalities. This study used the number of suicides from open data for 10 years from 2009 to 2018 to obtain shrinkage estimates of the standardized mortality ratio (SMR) using the Bayesian hierarchical model. We visualized and compared the regional disparities in suicide for each policy unit. For each gender and policy unit, adjacent regions had similar clusters of SMRs and positive spatial autocorrelation of global Moran’s I (p < 0.001 for each). Comparisons between each policy unit showed that even if the SMR was low for the prefectural units, there were regions with high SMRs in municipalities and secondary medical areas, and vice versa. It was found that assessing suicide solely on a prefecture-by-prefecture basis may overlook regional disparities in suicide. This research emphasizes the need to establish suicide indicators at the secondary medical or municipal level and execute individual suicide prevention interventions in neighboring communities. Prefectures can also play a role in developing collaborative cooperation between neighboring regions by acting as actors.

## Introduction

According to the World Health Organization, almost 800,000 people die by suicide each year worldwide, with a global age-standardized suicide rate of 10.5 per 100,000 people [1], making suicide prevention a global concern. Japan has the highest suicide rate among the G7 developed nations, at 14.3 per 100,000 people [1]. From 1998 to 2011, over 30,000 people in Japan died by suicide each year. While the suicide rate has continued to fall since then due to the effectiveness of suicide prevention initiatives, the number of suicides still surpasses 20,000 each year [2]. Previous research has linked suicide risk in Japan to economic recession and unemployment [2]. Still, socio-cultural and other factors are also thought to have a role [3], and many causes remain unknown. Understanding the features of suicide cases in Japan and taking countermeasures is a critical policy issue because Japan’s suicide rate remains high.

**Fig 1:**
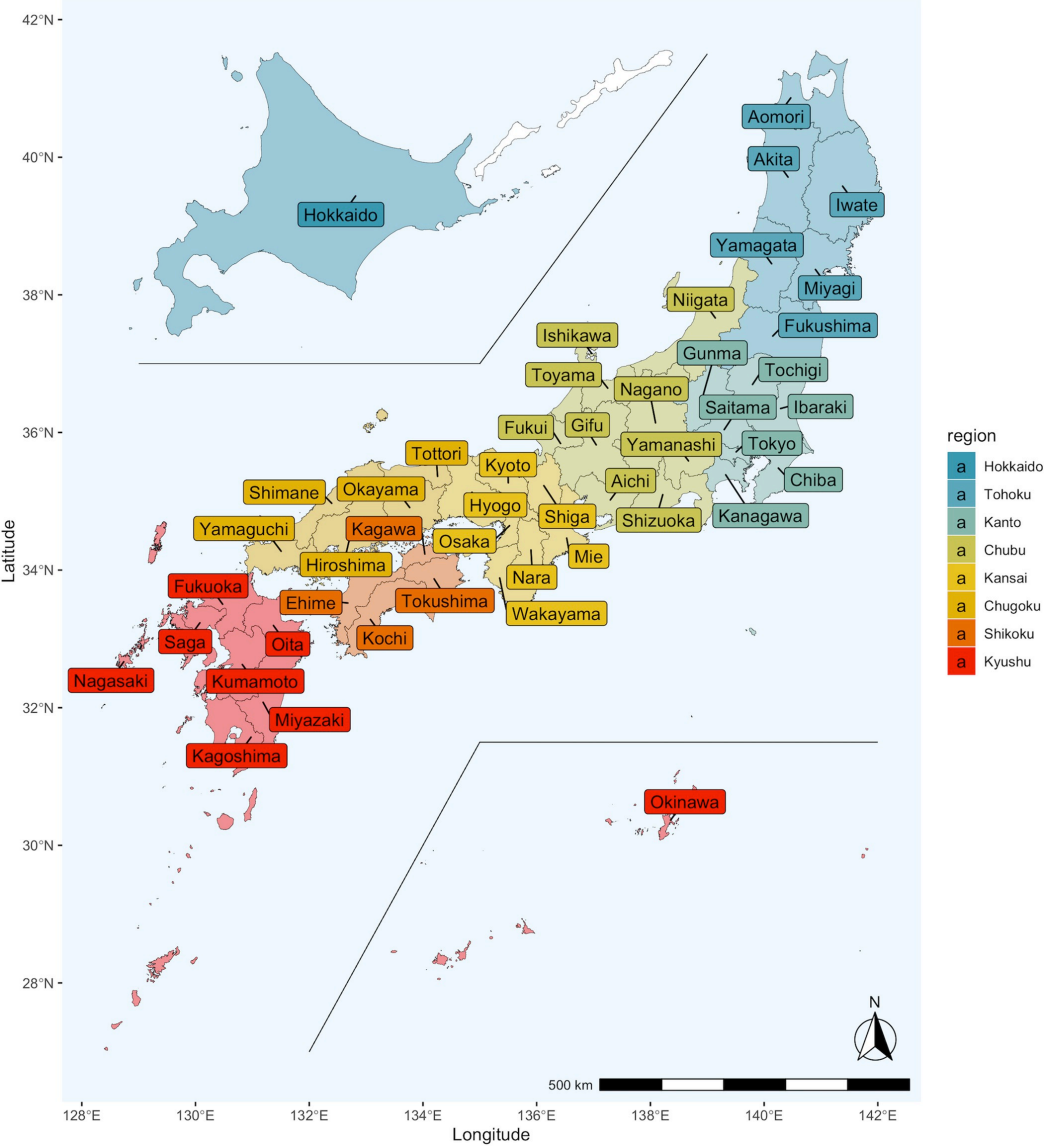
Map of Japan and prefectures. Japan has 47 prefectures divided into eight regions. These regions are not official administrative units but are used only for statistical and other purposes. The following map data (CC BY 4.0) was processed and used: Ministry of Land, Infrastructure, Transport and Tourism. National surveys of Japan—Administrative area data (in Japanese). 2019. Available from: https://nlftp.mlit.go.jp/ksj/gml/datalist/KsjTmplt-N03-v3_0.html.

The Japanese government system is divided into three levels: national, prefectural, and municipal, each of which forms a policy unit and works together. The national government consists of 47 prefectures (Fig 1). Each prefecture consists of many municipalities. As of October 2018, there were a total of 1,896 municipalities. Both prefectures and municipalities are obligated to formulate suicide prevention plans under the revised Basic Law on Suicide Prevention of April 2016 [4]. Furthermore, according to the Medical Service Law, there are policy units in Japan called secondary medical areas, defined as areas where general inpatient care is available. Three hundred thirty-five areas have been set up to provide efficient medical services as of January 2021 [5]. Studies on suicide have been conducted at each of the following regional levels: prefectures [6–12], secondary health care areas [13, 14], municipal towns and villages [15–20], and cities and villages in specific prefectures [21, 22]. It is critical to comprehensively assess the efficiency of suicide prevention measures in each administrative unit, such as prefectures, secondary health care areas, and municipalities, to assure the success of suicide prevention measures throughout Japan. However, it is unclear how these policy units’ efforts should be linked to one another and how they should be verified [23].

Except for a few studies [7, 15, 17, 20] that noted that suicide neighborhoods clustered in spatial contiguity, most studies did not investigate the impact of spatial proximity on neighborhood suicide. This is problematic because suicide is influenced not only by the individual but also by the surrounding context [24]. There has been some discussion around suicide resulting from a chain reaction of personal factors and a combination of community and social factors [25]. When considering effective countermeasures in line with local conditions, it is essential to clarify regional disparities in suicide, considering spatial continuity. In Japan, regional cluster suicides have been recorded using a spatial epidemiology technique called spatial scanning [15]. Previous studies using spatial regression models suggested a regional continuum of suicide in Japan, related to access to psychiatric care population density and cultural values. Still, no further information is available [7, 17, 20]. In addition, it is not clear whether it is better to use the policy unit of the prefecture, secondary medical area, or municipality when considering spatial continuity. Thus, clarifying the interconnectedness in the regional disparities in suicide among policy units can provide indicators for developing organic and effective suicide prevention plans in cooperation with each policy unit. Therefore, this study analyzed the regional characteristics of suicide at the municipal, secondary medical, and prefectural levels using open data on suicide and spatial statistical methods. While using data for a sufficient period, we examined the spatial continuity of each policy unit after solving the subregional problem by smoothing the SMRs with a model that assumes no spatial autocorrelation. We discussed the differences in visibility by mapping the SMRs for each policy unit.

## Material and methods

### Data source

The research was designed as an ecological study using nationwide suicide deaths and population data for 10 years from 2009 to 2018. The sample size of suicide deaths was taken from residential area data published annually [26]. We used data based on the Basic Resident Registration [27]. From the data on the number of suicides and the population, we calculated the standardized mortality ratio (SMR) of the number of suicides in each municipality. To visually map the SMR data, we used the national administrative area data [28], all of which are open to the public and easily accessible.

From the polygon data of administrative areas obtained from the National Land Numerical Information, we generated polygon data integrated into prefectures, secondary medical areas, and municipalities as of October 2018. Adjacency was defined as the boundaries of administrative regions touching each other. A total of 1887 municipalities, 344 secondary medical areas, and 47 prefectures were demarcated in Japan as of October 1, 2018.

### Measurements

SMRs in municipalities, secondary medical areas, and prefectures were defined as the primary outcomes in this study.

### Statistical analysis

#### Calculation of SMR

We calculated the SMR for the number of suicides from 2009 to 2018 for each of the municipalities, secondary medical areas, and prefectures. Three of the ordinance-designated cities (Okayama City, Sagamihara City, and Kumamoto City) were discovered to have reorganized their wards since 2009. In this survey, we counted each of them as cities. As a result, the total number of municipalities in 2018 was 1896, but it was aggregated to 1887 municipalities for the analysis [29].

Considering that the incidence of suicide is very low in small municipalities, we estimated the SMR using a Bayesian hierarchical model and using data of sufficient duration to ensure the number of cases to be analyzed [30–33]. First, we calculated the expected number of suicide deaths (*e*_*i*_) for municipality *i* = 1,…,1887 from the national age-specific suicide mortality rate for each year based on the population by age in 10-year increments and the number of suicides by age. Data on the number of suicides in unknown ages were excluded from the analysis in this study as it was impossible to calculate the expected values. Second, we modeled the number of suicide deaths *d*_*i*_ using the Poisson–Gamma model incorporating *e*_*i*_ calculated above and an unknown SMR parameter *θ*_*i*_: *d*_*i*_~Poisson(*e*_*i*_θ_*i*_) (first hierarchy) and *θ*_*i*_~*Gamma*(*α*, *β*) (second hierarchy), where (*α*, *β*) are shapes and scale (hyper-)parameters of the prior distribution of *θ*_*i*_ [30, 31]. One of the objectives of this study is to shrink the SMR and examine the spatial continuity of each policy unit in a model that assumes no spatial autocorrelation. Unlike previous studies, which allowed spatial autocorrelation [17] and used the weighted average and variance of expected suicide deaths in prefectures and secondary medical areas for (*α*, *β*) [34, 35], we assumed independence between *θ*_*i*_ so as not to induce spatial correlation among neighborhoods. Following previous studies [33], we chose exponential distribution with rate parameter of 1 as the prior distribution of *α* and Gamma (0.1, 1.0) for *β* (i.e., third hierarchy). By convention, the SMR was expressed with 100 *θ*_*i*_. Hence, if the number of observed and expected suicide deaths in the region *i* is the same (SMR = 100), then the risk of death in the region *i* is about the same as the average for Japan as a whole.

For data from secondary medical areas and prefectures, we combined the relevant municipalities and added up the values for each variable. The data for each municipality was added for 10 years, and the SMRs were recalculated as above [33]. For comparison, the “raw” SMR was also calculated as *d*_*i*_/*e*_*i*_.

#### Global Moran’s I

To quantitatively evaluate the spatial autocorrelation, the Global Moran’s I statistic [36, 37] was calculated as

$$I_{Global}=\frac{\sum_{i}\sum_{j}w_{ij}z_{i}.z_{j}/\sum_{i}\sum_{j}w_{ij}}{\sum_{i}z_{i}^{2}/n},$$

where $z_{i}=\theta_{i}-\bar{\theta }$ (the mean of *θ*), *w*_*ij*_ is the element of the spatial weight matrix, and *n* is the number of municipalities, secondary medical areas, or prefectures. For the weight matrix, we used an adjacency matrix, which includes 1 in (*i*, *j*)-element if regions *i* and *j* are adjacent and 0 otherwise. *I*_Global_ essentially measures and averages the covariance of the SMR of each region with the SMRs of its neighboring regions. In case there are many regions with SMRs like those of the neighboring areas (i.e., positive autocorrelation as a whole), *I*_Global_ takes a positive value between 0 and 1; the more significant the autocorrelation, the closer it is to 1. If many regions have different SMRs from neighboring areas, *I*_Global_ takes a negative value between 0 and -1.

#### Choropleth map

We used administrative area data and calculated SMR to create maps for visual consideration. For 10 years, the SMRs of municipalities, secondary medical areas, and prefectures were plotted on choropleth maps [38]. After that, we constructed bivariate choropleth maps of each SMR to look at the differences in SMR plots between policy units. The first and third quartiles of the SMR were established as the split points using known methods [39]. All analyses and mapping were conducted using R version 4.0.3 (R Project for Statistical Computing), RStan version 2.21.2, and the spdep R package [40].

### Ethics

Since only publicly available secondary data were used in this study, based on the Ethical Guidelines for Medical and Health Sciences Research Involving Human Subjects, we required no ethical review by the Ethical Review Committee [41].

## Results

**Fig 2:**
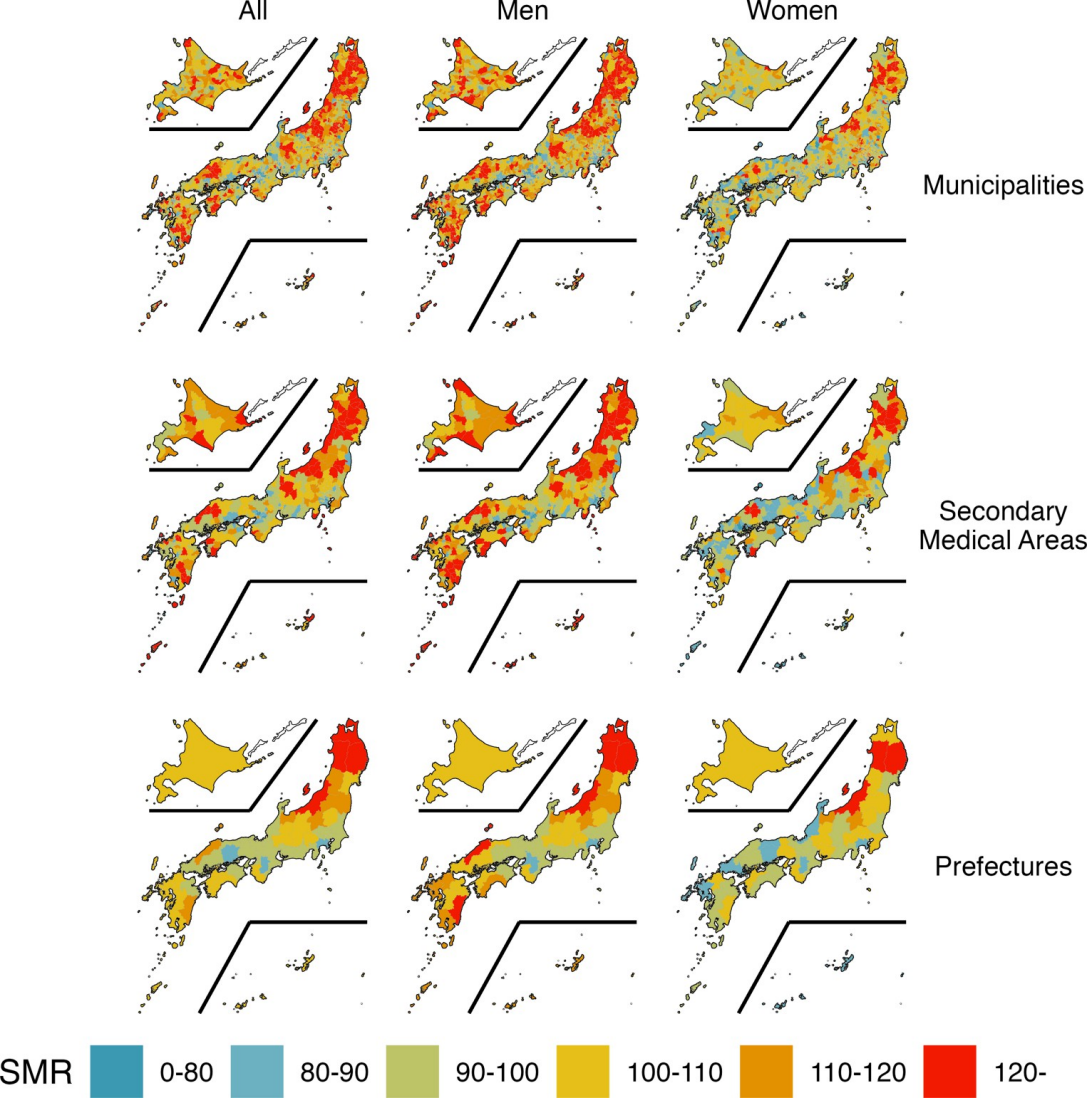
Geographical distribution of standardized death ratios for suicides. The standardized mortality ratios (SMRs) for all ages in 1887 municipalities, 334 secondary medical areas, and 47 prefectures in Japan during 2009–2018 were calculated and plotted. The plots were divided into facets by region size on the vertical axis and gender on the horizontal axis. The following map data (CC BY 4.0) was processed and used: Ministry of Land, Infrastructure, Transport and Tourism. National surveys of Japan—Administrative area data (in Japanese). 2019. Available from: https://nlftp.mlit.go.jp/ksj/gml/datalist/KsjTmplt-N03-v3_0.html.

There was a total of 258,497 suicides (178,447 men, or 69% of the total, and 80,050 women, or 31%) in the 10 years from 2009 to 2018, excluding cases with unknown ages. Fig 2 shows the choropleth map of the geographic distribution of SMRs for suicides across all ages by municipality, secondary care area, prefecture, and gender. The prefecture map shows, we find that the SMR tends to be high in the Tohoku region, northern Chubu region, northern Chugoku region, and southern Kyushu. However, the SMR in secondary medical care areas and municipalities was distributed in a patchwork manner, and regions with high SMRs were identified in addition to the areas mentioned above.

Table 1 shows the number of suicides, expected suicide deaths, and SMRs before (non-Bayesian) and after Bayesian estimation. Wilcoxon’s rank-sum test showed no difference between the non-Bayesian SMR and Bayesian SMR, except in the woman of municipalities variable.

**Table 1:** Number of suicides, expected suicide deaths, and standardized death ratios.

**Variable**	All^*a*^	Men^*a*^	Women^*a*^
	p-value^*c*^	p-value^*c*^	p-value^*c*^
**Municipalities**			
**N**	1,887	1,887	1,887
**Number of suicide deaths**	64 (23, 168)	45 (16, 120)	19 (7, 50)
**Expected suicide deaths**	62 (20, 165)	43 (14, 115)	20 (6, 51)
**SMR (non-Bayesian)**	103 (89, 121)	105 (88, 125)	98 (79, 118)
**SMR** ^***b***^	104 (95, 113)	105 (95, 115)	99 (93, 106)
	> 0.9	0.9	0.004
**Secondary medical areas**			
**N**	344	344	344
**Number of suicide deaths**	502 (262, 985)	361 (184, 686)	145 (73, 302)
**Expected suicide deaths**	471 (221, 963)	319 (150, 672)	145 (70, 294)
**SMR (non-Bayesian)**	105 (95, 114)	107 (95, 120)	98 (89, 110)
**SMR** ^***b***^	105 (95, 114)	107 (96, 119)	98 (91, 108)
	> 0.9	> 0.9	0.5
**Prefectures**			
**N**	47	47	47
**Number of suicide deaths**	3,503 (2,457, 5,806)	2,469 (1,697, 4,028)	1,042 (737, 1,730)
**Expected suicide deaths**	3,477 (2,324, 5,454)	2,311 (1,566, 3,714)	1,125 (740, 1,713)
**SMR (non-Bayesian)**	100 (98, 109)	105 (97, 112)	99 (91, 105)
**SMR** ^***b***^	100 (98, 109)	105 (97, 112)	99 (91, 105)
	> 0.9	> 0.9	> 0.9

Notes: SMR: standardized mortality ratio

^*a*^ Median (IQR)

^*b*^ SMRs were calculated from the number of suicide deaths and the expected value of suicide deaths, respectively, using the hierarchical Bayesian model

^*c*^ Wilcoxon rank sum test between non-Bayesian and Bayesian SMR.

Table 2 presents the global Moran’s I statistics obtained for each SMR. A positive spatial correlation was observed for each regional unit. Global Moran’s I tended to have higher Bayesian SMR than non-Bayesian SMR. In each municipality, secondary medical area, and prefecture, the Global Moran’s I tended to be smaller for women than for men or both genders.

**Table 2:** Global Moran’s I.

**Variable**	Global Moran’s I^*a*^			
	**SMR (non-Bayesian)**	p-value^*b*^	**SMR**	p-value^*b*^
**Municipalities**				
**All**	0.22	< 0.001	0.42	< 0.001
**Men**	0.21	< 0.001	0.44	< 0.001
**Women**	0.11	< 0.001	0.25	< 0.001
**Secondary medical areas**				
**All**	0.37	< 0.001	0.39	< 0.001
**Men**	0.39	< 0.001	0.43	< 0.001
**Women**	0.27	< 0.001	0.31	< 0.001
**Prefectures**				
**All**	0.45	< 0.001	0.45	< 0.001
**Men**	0.48	< 0.001	0.49	< 0.001
**Women**	0.33	< 0.001	0.33	< 0.001

Notes: SMR: standardized mortality ratio

^*a*^ Global Moran’s I statistic for standardized death ratios for suicide from 2009 to 2018

^*b*^ Moran’s I test under randomization.

**Fig 3:**
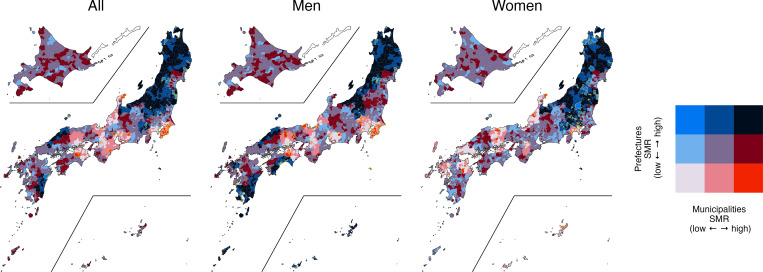
Bivariate map between municipalities and prefectures. SMR: standardized mortality ratio; geographic distribution was visualized based on the tertiles of each region. The following map data (CC BY 4.0) was processed and used: Ministry of Land, Infrastructure, Transport and Tourism. National surveys of Japan—Administrative area data (in Japanese). 2019. Available from: https://nlftp.mlit.go.jp/ksj/gml/datalist/KsjTmplt-N03-v3_0.html.

**Fig 4:**
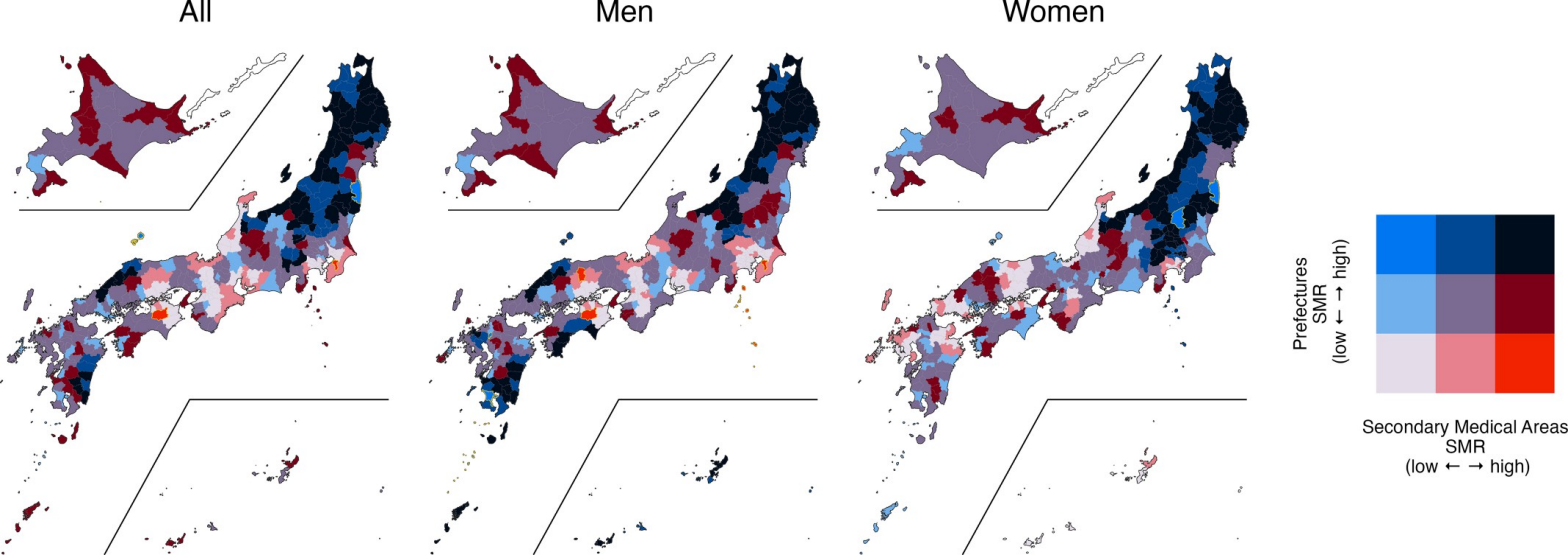
Bivariate map between secondary medical areas and prefectures. SMR: standardized mortality ratio; geographic distribution was visualized based on the tertiles of each region. The following map data (CC BY 4.0) was processed and used: Ministry of Land, Infrastructure, Transport and Tourism. National surveys of Japan—Administrative area data (in Japanese). 2019. Available from: https://nlftp.mlit.go.jp/ksj/gml/datalist/KsjTmplt-N03-v3_0.html.

**Fig 5:**
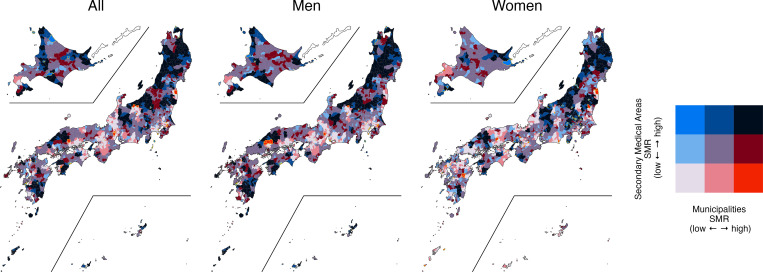
Bivariate map between municipalities and secondary medical areas. SMR: standardized mortality ratio; geographic distribution was visualized based on the tertiles of each region. The following map data (CC BY 4.0) was processed and used: Ministry of Land, Infrastructure, Transport and Tourism. National surveys of Japan—Administrative area data (in Japanese). 2019. Available from: https://nlftp.mlit.go.jp/ksj/gml/datalist/KsjTmplt-N03-v3_0.html.

Table 3 shows the SMRs for each policy unit, divided into low, medium, and high in the first and third quartiles. The same regions were classified by a municipality and secondary medical area, municipality and prefecture, and secondary medical area and prefecture. Figs 3–5 show the bivariate map visualizing the geographic distribution based on the tertile of each region [39].

**Table 3:** Comparison of tertile by area.

**Variable**	All^*a*^	Men^*a*^	Women^*a*^
**Tertile** ^*b*^ **, Prefectures—Municipalities, N = 1,887**			
High-Low	26 (1.4%)	27 (1.4%)	60 (3.2%)
High-Middle	165 (8.7%)	174 (9.2%)	189 (10%)
High-High	185 (9.8%)	187 (9.9%)	216 (11%)
Middle-Low	226 (12%)	188 (10.0%)	275 (15%)
Middle-Middle	574 (30%)	521 (28%)	603 (32%)
Middle-High	247 (13%)	240 (13%)	231 (12%)
Low-Low	220 (12%)	257 (14%)	137 (7.3%)
Low-Middle	204 (11%)	248 (13%)	151 (8.0%)
Low-High	40 (2.1%)	45 (2.4%)	25 (1.3%)
**Tertile** ^*b*^ **, SMA** ^c^ **—Municipalities, N = 1,887**			
High-Low	16 (0.8%)	9 (0.5%)	29 (1.5%)
High-Middle	158 (8.4%)	157 (8.3%)	163 (8.6%)
High-High	274 (15%)	266 (14%)	262 (14%)
Middle-Low	176 (9.3%)	166 (8.8%)	218 (12%)
Middle-Middle	617 (33%)	614 (33%)	571 (30%)
Middle-High	183 (9.7%)	190 (10%)	187 (9.9%)
Low-Low	280 (15%)	297 (16%)	225 (12%)
Low-Middle	168 (8.9%)	172 (9.1%)	209 (11%)
Low-High	15 (0.8%)	16 (0.8%)	23 (1.2%)
**Tertile** ^*b*^ **, Prefectures—SMA** ^c^ **, N = 344**			
High-Low	2 (0.6%)	2 (0.6%)	2 (0.6%)
High-Middle	32 (9.3%)	30 (8.7%)	32 (9.3%)
High-High	45 (13%)	49 (14%)	54 (16%)
Middle-Low	35 (10%)	26 (7.6%)	41 (12%)
Middle-Middle	115 (33%)	113 (33%)	114 (33%)
Middle-High	39 (11%)	33 (9.6%)	32 (9.3%)
Low-Low	49 (14%)	58 (17%)	43 (12%)
Low-Middle	25 (7.3%)	29 (8.4%)	26 (7.6%)
Low-High	2 (0.6%)	4 (1.2%)	0 (0%)

Notes

^*a*^ n (%)

^*b*^ standardized death ratios (SMRs) of suicide were divided into the first and third quartiles (low, middle, and high)

^c^ SMA, secondary medical areas

The dichotomy between prefectures and municipalities is shown in Table 3 and Fig 3. The SMR of the prefecture was low and that of the municipality was high (Low-High) in 40 regions (2.1%) for both genders, 45 regions (2.4%) for men, and 25 regions (1.3%) for women. Red zones (Low-High) were identified for both genders and men, mainly in Chiba Prefecture. By contrast, the SMR of the prefectures was high and that of the municipalities was low (High-Low) in 26 regions (1.4%) for both genders, 45 regions (1.4%) for men, and 60 regions (3.2%) for women. The blue region (High-Low), concentrated in the Tohoku region and parts of the Kanto and Chubu regions, caters to more women.

Compared with the bivariate map [39] of secondary medical area and prefecture (Fig 4), the general trend is the same as that of the map of municipality and prefecture. In the bivariate map of municipalities and secondary medical care areas (Fig 5), new red zones (Low-High) and blue zones (High-Low) were identified. For example, a red zone (Low-High) for men was identified in a part of Hiroshima Prefecture, and one for women was identified in Miyazaki and Miyagi prefectures. A new blue zone (High-Low) was confirmed for women in the eastern part of Hokkaido.

## Discussion

This study is the first to visualize and compare regional disparities in suicide by policy units in municipalities, secondary medical areas, and prefectures in Japan. There was a positive spatial autocorrelation in the SMR of suicide in adjacent regions for each gender and policy unit. In comparing each policy unit, even within some prefectures with low SMRs, high SMRs were observed at the municipality and secondary medical area levels. At the same time, the opposite (low SMR in municipalities but high in prefectures) also holds. To solve sub-regional estimation, we used the number of suicides pooled over 10 years from 2009 to 2018. We obtained a shrinkage estimate of SMR that minimizes the effect of the population size using the Bayesian hierarchical model. Even with 10 years of data, there was a statistical difference between the raw SMR and the Bayesian SMR among women in the municipality, which was necessary to solve the subregional problem. Several studies have examined the geographic continuity and clustering of suicides in Japan [7, 17, 20], but this is the first study to visualize and compare the number of suicides by the municipality, secondary medical area, and prefecture.

This study first examined the differences in the visualization of SMRs by policy unit by combining municipalities, secondary medical care areas, and prefectures to visualize their spatial extent (Table 3, Figs 3–5). Even though the SMR for the prefecture was low, regions with high SMRs in municipalities and secondary medical areas. This indicates that if we analyze suicide factors by prefecture, we could overlook areas with high suicide rates. Thus, additional analysis is necessary not only for prefectures but also for smaller regions, such as municipalities and secondary medical areas based on the results of past studies conducted at the prefectural level on suicide and various factors—temperature [12], increased expenditures by local governments [42], social leisure activities [10], economic indicators, such as average annual income and income disparity [11], occupations [8], marriage rate, and full employment rate [9]. On the contrary, even if the SMR for the entire prefecture was high, there were areas where the SMR was low, such as in municipalities or secondary medical care areas. These regions within the same prefecture may be doing well in suicide prevention, reflecting the specific problems faced by those municipalities and the unique efforts of each regional administrative unit in mitigating them. By comparing differences in suicide-related factors in neighboring municipalities within the same prefecture, it may be possible to find influential factors in preventing suicide.

Second, we plotted the distribution of suicide SMRs for each policy unit on a map (Fig 2). For each gender and policy unit, neighboring regions showed similar clusters of SMRs, and the Global Moran’s I also showed positive spatial autocorrelation for each (Table 2). The finding that there is spatial continuity in suicide is consistent with the literature to date [7, 17, 20]. Previous studies using spatial regression models in Japan have revealed a regional continuum of suicide at the municipal level in Japan, which is associated with population density, access to mental health care, and cultural values [7, 17, 20]. Other factors pointed out include access to means of suicide and land slope at the municipal level, education level and household structure at the secondary health care area level, and socioeconomics at the prefectural level, but these studies do not include adjacent data in their analysis, so interpretation of whether there is continuity must be made carefully.

These results show spatial continuity and suggest that we can delve deeper into the factors contributing to suicide by highlighting overlooked areas. We also found regions with high suicide rates and areas with low suicide rates, even adjacent. It is assumed that these regions have similar background structures such as geography and socioeconomics, and it would be easier to examine policy differences as a more case-control study. We believe that comparing suicide prevention with neighboring regions may provide insight into the protective factors of suicide. Policymakers and researchers are encouraged to conduct higher resolution studies based on coarse regional trends. If there is no continuum of suicide in an area, it would be worthwhile to investigate what is acting as a protective factor. Since prefectures have a specific form of administrative authority over municipalities and secondary medical care areas [43], they can also be expected to develop cooperation among neighboring regions by acting as an actor. The secondary medical or municipal level analysis is superior to that at the prefectural level regarding not overlooking regional disparities in suicide. However, we found that the smaller the region, the more limited the data can be obtained. When conducting regional correlation studies at the municipal level, it is desirable to use analytical methods, such as multilevel analysis and Bayesian hierarchical models, which simultaneously treat the status of prefectures and secondary medical areas as variables. In the future, studies using Spatio-temporal statistical methods could be helpful for more effective and specific suicide prevention.

This study has several limitations. First, we used 10 years of data to solve the sub-regional problem. Over this period, suicide prevention efforts and the socio-economic context of the region could have changed, and the impact of these changes might not have been adequately estimated. However, there was a statistical difference between the raw SMRs and the Bayesian SMRs of women in the municipality, even with 10 years of data. Because the small number of years may cause errors in mapping the regions, we did not conduct a time series analysis and only summarized the data for 10 years. In addition, during the survey period, the Japanese economy was recovering moderately, driven mainly by domestic demand, and economic waves were thought to be minor [44]. Second, the analysis was conducted for all age groups. Different age groups may have different risk factors for suicide. Third, it is debatable whether it is appropriate to divide the SMR into the first and third quartiles in creating the bivariate map. This study used an existing method to divide the data into the first and third quartiles [39]. It is important to note that the artificial division of data may overlook or accentuate regional disparities in suicide.

This study visualized regional disparities in suicide at the policy level in municipalities, secondary medical areas, and prefectures across Japan, showing spatial continuity and revealing regional differences in suicide that were overlooked in the prefectures. Further analysis clarifying the preventive factors at the regional and societal levels would contribute to evaluating the effects and evidence of suicide prevention measures in each policy unit.

## Supporting information

S1 File (DOCX)
