# Supplementary material for: Spatial statistical analysis of regional disparities in suicide among policy units in Japan: Using the Bayesian hierarchical model
Source: PLOS Glob Public Health. 2022 Aug 15;2(8):e0000271. doi: 10.1371/journal.pgph.0000271 (PMC10021712; doi:10.1371/journal.pgph.0000271)
Supplement: S1 File — (DOCX) [file pgph.0000271.s001.docx]

**Table A. Prefectures with high suicide standardized mortality ratio (SMR) and municipalities with low SMR.**

| Prefecture | Prefecture SMR ***^a^*** | Municipality | Municipality SMR ***^a^*** |
| --- | --- | --- | --- |
| Aomori | 120.5 | Ajigasawa-cho, Nishitsugaru-gun | 78.5 |
| Aomori | 120.5 | Noheji-cho, Kamikita-gun | 93.8 |
| Aomori | 120.5 | Oma Town, Shimokita District | 89.4 |
| Iwate | 128.9 | Rikuzentakata City | 94.0 |
| Iwate | 128.9 | Otsuchi-cho, Kamihei-gun | 92.3 |
| Yamagata | 111.6 | Yamagata City | 90.6 |
| Yamagata | 111.6 | Higashine City | 89.1 |
| Yamagata | 111.6 | Nakayama-cho, Higashimurayama-gun | 88.2 |
| Yamagata | 111.6 | Nishikawa-cho, Nishimurayama-gun | 89.2 |
| Fukushima | 110.7 | Koori Town, Date District | 92.9 |
| Fukushima | 110.7 | Naraha Town, Futaba District | 74.0 |
| Fukushima | 110.7 | Tomioka Town, Futaba District | 62.9 |
| Fukushima | 110.7 | Okuma Town, Futaba District | 74.1 |
| Fukushima | 110.7 | Futaba Town, Futaba District | 71.2 |
| Fukushima | 110.7 | Namie Town, Futaba District | 66.8 |
| Fukushima | 110.7 | Katsurao Village, Futaba District | 92.4 |
| Fukushima | 110.7 | Iitate Village, Soma District | 84.4 |
| Tochigi | 110.0 | Tochigi City | 93.0 |
| Tochigi | 110.0 | Shimotsuke City | 87.0 |
| Gunma | 113.6 | Kawaba Village, Tone District | 93.5 |
| Gunma | 113.6 | Itakura-cho, Ora-gun | 86.3 |
| Toyama | 112.9 | Namerikawa City | 91.5 |
| Yamanashi | 108.9 | Koshu City | 86.7 |
| Yamanashi | 108.9 | Nishikatsura Town, Minamitsuru District | 90.9 |
| Shimane | 114.7 | Okinoshima Town, Oki District | 92.8 |
| Miyazaki | 116.5 | Nobeoka City | 91.5 |

***^a^*** SMR: Standardized mortality ratio

**Table B. Prefectures with high suicide standardized mortality ratio (SMR) and municipalities with low SMR in men.**

| Prefecture | Prefecture SMR ***^a^*** | Municipality | Municipality SMR ***^a^*** |
| --- | --- | --- | --- |
| Aomori | 129.4 | Ajigasawa-cho, Nishitsugaru-gun | 81.1 |
| Aomori | 129.4 | Oma Town, Shimokita District | 91.6 |
| Iwate | 128.8 | Rikuzentakata City | 89.8 |
| Yamagata | 114.0 | Yamagata City | 92.2 |
| Yamagata | 114.0 | Sagae City | 89.7 |
| Yamagata | 114.0 | Higashine City | 94.5 |
| Yamagata | 114.0 | Yamanobe-cho, Higashimurayama-gun | 94.6 |
| Yamagata | 114.0 | Nakayama-cho, Higashimurayama-gun | 84.3 |
| Yamagata | 114.0 | Kahoku Town, Nishimurayama District | 95.1 |
| Yamagata | 114.0 | Nishikawa-cho, Nishimurayama-gun | 90.3 |
| Gunma | 112.6 | Yoshioka Town, Kitagunma District | 94.4 |
| Gunma | 112.6 | Tamamura Town, Sawa District | 94.3 |
| Gunma | 112.6 | Takayama Village, Agatsuma District | 95.0 |
| Gunma | 112.6 | Itakura-cho, Ora-gun | 87.6 |
| Gunma | 112.6 | Ora-cho, Ora-gun | 85.9 |
| Niigata | 123.6 | Akiha-ku, Niigata City | 94.7 |
| Kochi | 114.2 | Tosa City | 88.3 |
| Kochi | 114.2 | Konan City | 91.6 |
| Kochi | 114.2 | Susaki City | 86.0 |
| Saga | 112.5 | Kiyama-cho, Miyaki-gun | 89.2 |
| Saga | 112.5 | Miyaki Town, Miyaki District | 91.3 |
| Saga | 112.5 | Kashima City | 85.5 |
| Kagoshima | 116.4 | Kagoshima City | 92.3 |
| Kagoshima | 116.4 | Ibusuki | 91.6 |
| Kagoshima | 116.4 | Nagashima Town, Izumi District | 92.6 |
| Okinawa | 117.7 | Ginoza Village, Kunigami District | 95.1 |
| Okinawa | 117.7 | Yonabaru Town, Shimajiri District | 92.1 |

***^a^*** SMR: Standardized mortality ratio

**Table C. Prefectures with high suicide standardized mortality ratio (SMR) and municipalities with low SMR in women.**

| Prefecture | Prefecture SMR ***^a^*** | Municipality | Municipality SMR ***^a^*** |
| --- | --- | --- | --- |
| Aomori | 105.7 | Hirosaki City | 90.9 |
| Aomori | 105.7 | Kuroishi City | 89.7 |
| Aomori | 105.7 | Aomori City | 92.7 |
| Aomori | 105.7 | Tsugaru City | 91.2 |
| Aomori | 105.7 | Ajigasawa-cho, Nishitsugaru-gun | 91.1 |
| Aomori | 105.7 | Fukaura Town, Nishitsugaru District | 91.7 |
| Aomori | 105.7 | Noheji-cho, Kamikita-gun | 86.9 |
| Iwate | 129.9 | Otsuchi-cho, Kamihei-gun | 90.6 |
| Akita | 130.0 | Ogata Village, Minamiakita District | 93.0 |
| Akita | 130.0 | Yuzawa City | 92.7 |
| Yamagata | 106.2 | Yamagata City | 90.2 |
| Yamagata | 106.2 | Kaminoyama City | 91.1 |
| Yamagata | 106.2 | Tendo City | 92.3 |
| Yamagata | 106.2 | Higashine City | 84.5 |
| Yamagata | 106.2 | Yonezawa City | 84.3 |
| Yamagata | 106.2 | Iide Town, Nishiokitama District | 90.4 |
| Fukushima | 105.9 | Motomiya City | 80.4 |
| Fukushima | 105.9 | Yabuki-cho, Nishishirakawa-gun | 84.4 |
| Fukushima | 105.9 | Hirono Town, Futaba District | 89.8 |
| Fukushima | 105.9 | Naraha Town, Futaba District | 91.7 |
| Fukushima | 105.9 | Tomioka Town, Futaba District | 76.0 |
| Fukushima | 105.9 | Okuma Town, Futaba District | 90.0 |
| Fukushima | 105.9 | Futaba Town, Futaba District | 90.4 |
| Fukushima | 105.9 | Namie Town, Futaba District | 83.2 |
| Fukushima | 105.9 | Iitate Village, Soma District | 90.6 |
| Fukushima | 105.9 | Inawashiro Town, Yama District | 91.2 |
| Fukushima | 105.9 | Yugawa Village, Kawanuma District | 92.6 |
| Tochigi | 109.5 | Nikko City | 85.0 |
| Tochigi | 109.5 | Moka City | 87.2 |
| Tochigi | 109.5 | Tochigi City | 88.1 |
| Tochigi | 109.5 | Shimotsuke City | 89.8 |
| Gunma | 113.1 | Shinto Village, Kitagunma District | 90.1 |
| Gunma | 113.1 | Fujioka City | 90.7 |
| Gunma | 113.1 | Kawaba Village, Tone District | 92.5 |
| Gunma | 113.1 | Itakura-cho, Ora-gun | 92.7 |
| Saitama | 107.9 | Toda City | 86.6 |
| Saitama | 107.9 | Wako city | 92.8 |
| Saitama | 107.9 | Miyoshi-cho, Iruma-gun | 86.5 |
| Saitama | 107.9 | Yashio City | 90.2 |
| Saitama | 107.9 | Higashimatsuyama City | 84.7 |
| Saitama | 107.9 | Namegawa-cho, Hiki-gun | 89.6 |
| Saitama | 107.9 | Yoshimi-cho, Hiki-gun | 85.8 |
| Saitama | 107.9 | Sugito-cho, Kitakatsushika-gun | 88.3 |
| Saitama | 107.9 | Yorii-cho, Osato-gun | 91.8 |
| Tokyo | 109.4 | Bunkyo-ku | 87.8 |
| Tokyo | 109.4 | Ota-ku | 92.5 |
| Tokyo | 109.4 | Setagaya-ku | 91.6 |
| Tokyo | 109.4 | Hinode-machi, Nishitama-gun | 90.7 |
| Tokyo | 109.4 | Inagi City | 83.2 |
| Tokyo | 109.4 | Mitaka City | 90.6 |
| Tokyo | 109.4 | Fuchu City | 87.3 |
| Tokyo | 109.4 | Kiyose City | 87.2 |
| Niigata | 128.5 | Tainai City | 91.1 |
| Toyama | 115.0 | Asahi-cho, Shimoniikawa-gun | 90.4 |
| Toyama | 115.0 | Namerikawa City | 81.0 |
| Yamanashi | 105.5 | Nirasaki City | 89.5 |
| Yamanashi | 105.5 | Chuo City | 83.0 |
| Yamanashi | 105.5 | Koshu City | 91.4 |
| Yamanashi | 105.5 | Nambu Town, Minamikoma District | 91.3 |
| Yamanashi | 105.5 | Uenohara City | 92.6 |

***^a^*** SMR: Standardized mortality ratio

**Table D. Prefectures with low suicide standardized mortality ratio (SMR) and municipalities with high SMR.**

| Prefecture | Prefecture SMR ***^a^*** | Municipality | Municipality SMR ***^a^*** |
| --- | --- | --- | --- |
| Chiba | 97.5 | Yachimata City | 116.3 |
| Chiba | 97.5 | Tomisato City | 119.3 |
| Chiba | 97.5 | Sakae Town, Imba District | 117.1 |
| Chiba | 97.5 | Sosa City | 116.1 |
| Chiba | 97.5 | Togane City | 119.6 |
| Chiba | 97.5 | Katsuura City | 116.8 |
| Chiba | 97.5 | Sanmu City | 131.0 |
| Chiba | 97.5 | Isumi City | 113.4 |
| Chiba | 97.5 | Mutsuzawa-cho, Chosei-gun | 124.0 |
| Chiba | 97.5 | Chosei-mura, Chosei-gun | 116.1 |
| Chiba | 97.5 | Shirako-cho, Chosei-gun | 120.5 |
| Chiba | 97.5 | Otaki-cho, Isumi-gun | 143.7 |
| Chiba | 97.5 | Minamiboso City | 115.0 |
| Chiba | 97.5 | Kimitsu City | 117.4 |
| Chiba | 97.5 | Ichihara City | 118.4 |
| Kanagawa | 86.1 | Kawasaki-ku, Kawasaki City | 131.9 |
| Kanagawa | 86.1 | Miura City | 113.3 |
| Kanagawa | 86.1 | Matsuda Town, Ashigarakami District | 118.1 |
| Kanagawa | 86.1 | Yugawara-machi, Ashigarashimo-gun | 115.8 |
| Kanagawa | 86.1 | Naka-ku, Yokohama City | 129.2 |
| Ishikawa | 95.7 | Suzu City | 128.3 |
| Ishikawa | 95.7 | Noto-cho, Hosu-gun | 118.6 |
| Fukui | 91.3 | Takahama Town, Oi District | 123.5 |
| Aichi | 97.0 | Shitara-cho, Kitashitara-gun | 125.2 |
| Aichi | 97.0 | Nakamura-ku, Nagoya City | 129.3 |
| Aichi | 97.0 | Naka-ku, Nagoya City | 141.9 |
| Aichi | 97.0 | Atsuta-ku, Nagoya City | 116.2 |
| Aichi | 97.0 | Nakagawa-ku, Nagoya City | 115.7 |
| Aichi | 97.0 | Minato-ku, Nagoya City | 120.7 |
| Mie | 95.1 | Inabe City | 123.0 |
| Mie | 95.1 | Kihoku Town, Kitamuro District | 115.3 |
| Kyoto | 91.2 | Ide-cho, Tsuzuki-gun | 124.3 |
| Osaka | 92.4 | Taisho-ku, Osaka City | 115.2 |
| Osaka | 92.4 | Naniwa-ku, Osaka City | 170.6 |
| Osaka | 92.4 | Ikuno-ku, Osaka City | 148.1 |
| Osaka | 92.4 | Nishinari-ku, Osaka City | 199.0 |
| Osaka | 92.4 | Hirano-ku, Osaka City | 117.4 |
| Osaka | 92.4 | Chuo-ku, Osaka City | 123.5 |
| Tokushima | 90.2 | Tsurugi Town, Mima District | 118.1 |
| Tokushima | 90.2 | Miyoshi City | 138.4 |

***^a^*** SMR: Standardized mortality ratio

**Table E. Prefectures with low suicide standardized mortality ratio (SMR) and municipalities with high SMR in men.**

| Prefecture | Prefecture SMR ***^a^*** | Municipality | Municipality SMR ***^a^*** |
| --- | --- | --- | --- |
| Saitama | 95.0 | Namegawa-cho, Hiki-gun | 126.7 |
| Saitama | 95.0 | Higashi-Chichibu-mura, Chichibu-gun | 115.5 |
| Saitama | 95.0 | Misato Town, Kodama District | 123.1 |
| Saitama | 95.0 | Kamikawa-cho, Kodama-gun | 117.4 |
| Saitama | 95.0 | Minano-cho, Chichibu-gun | 122.1 |
| Chiba | 94.7 | Yachimata City | 119.5 |
| Chiba | 94.7 | Tomisato City | 118.4 |
| Chiba | 94.7 | Choshi City | 116.8 |
| Chiba | 94.7 | Togane City | 121.3 |
| Chiba | 94.7 | Katsuura City | 122.3 |
| Chiba | 94.7 | Sanmu City | 128.2 |
| Chiba | 94.7 | Chosei-mura, Chosei-gun | 127.8 |
| Chiba | 94.7 | Shirako-cho, Chosei-gun | 124.9 |
| Chiba | 94.7 | Otaki-cho, Isumi-gun | 135.3 |
| Chiba | 94.7 | Minamiboso City | 121.5 |
| Chiba | 94.7 | Kimitsu City | 115.3 |
| Chiba | 94.7 | Ichihara City | 119.6 |
| Tokyo | 94.2 | Taito-ku | 127.2 |
| Tokyo | 94.2 | Shinjuku-ku | 115.4 |
| Tokyo | 94.2 | Hinohara Village, Nishitama District | 115.4 |
| Tokyo | 94.2 | Okutama Town, Nishitama District | 121.8 |
| Tokyo | 94.2 | Higashimurayama City | 121.2 |
| Tokyo | 94.2 | Hachijo Town | 221.9 |
| Kanagawa | 83.3 | Kawasaki-ku, Kawasaki City | 125.5 |
| Kanagawa | 83.3 | Miura City | 118.1 |
| Kanagawa | 83.3 | Matsuda Town, Ashigarakami District | 124.2 |
| Kanagawa | 83.3 | Naka-ku, Yokohama | 120.8 |
| Fukui | 96.4 | Mihama-cho, Mikata-gun | 118.9 |
| Fukui | 96.4 | Takahama Town, Oi District | 133.0 |
| Aichi | 93.6 | Mihama-cho, Chita-gun | 115.3 |
| Aichi | 93.6 | Shitara-cho, Kitashitara-gun | 127.1 |
| Aichi | 93.6 | Nakamura-ku, Nagoya City | 120.1 |
| Aichi | 93.6 | Naka-ku, Nagoya City | 118.1 |
| Aichi | 93.6 | Minato-ku, Nagoya City | 117.9 |
| Kyoto | 90.9 | Kyotango City | 115.9 |
| Kyoto | 90.9 | Ide-cho, Tsuzuki-gun | 143.7 |
| Osaka | 89.9 | Naniwa-ku, Osaka City | 149.8 |
| Osaka | 89.9 | Ikuno-ku, Osaka City | 135.0 |
| Osaka | 89.9 | Nishinari-ku, Osaka City | 199.7 |
| Okayama | 92.4 | Maniwa City | 120.9 |
| Okayama | 92.4 | Mimasaka City | 115.6 |
| Tokushima | 90.6 | Tsurugi Town, Mima District | 115.4 |
| Tokushima | 90.6 | Miyoshi City | 140.3 |
| Kagawa | 93.7 | Sakaide City | 124.4 |
| Kagawa | 93.7 | Ayagawa-cho, Ayauta-gun | 117.4 |

***^a^*** SMR: Standardized mortality ratio

**Table F. Prefectures with low suicide standardized mortality ratio (SMR) and municipalities with high SMR in women.**

| Prefecture | Prefecture SMR ***^a^*** | Municipality | Municipality SMR ***^a^*** |
| --- | --- | --- | --- |
| Kanagawa | 89.5 | Kawasaki-ku, Kawasaki City | 123.1 |
| Kanagawa | 89.5 | Atsugi City | 111.2 |
| Kanagawa | 89.5 | Zama City | 114.8 |
| Kanagawa | 89.5 | Hakone-machi, Ashigarashimo-gun | 111.9 |
| Kanagawa | 89.5 | Yugawara-machi, Ashigarashimo-gun | 111.4 |
| Kanagawa | 89.5 | Naka-ku, Yokohama | 129.1 |
| Ishikawa | 89.8 | Kaga City | 109.7 |
| Ishikawa | 89.8 | Suzu City | 111.9 |
| Nara | 85.0 | Yamazoe Village, Yamabe District | 113.6 |
| Nara | 85.0 | Ando Town, Ikoma District | 109.2 |
| Nara | 85.0 | Kammaki-cho, Kitakatsuragi-gun | 109.0 |
| Nara | 85.0 | Gose City | 110.4 |
| Tottori | 83.6 | Kurayoshi City | 107.8 |
| Tottori | 83.6 | Misasa Town, Tohaku District | 109.5 |
| Okayama | 83.9 | Kibichuo Town, Kaga District | 109.1 |
| Yamaguchi | 87.1 | Hirao Town, Kumage District | 109.3 |
| Saga | 88.5 | Taku City | 106.7 |
| Saga | 88.5 | Yoshinogari Town, Kanzaki District | 111.5 |
| Nagasaki | 86.9 | Togitsu-cho, Nishisonogi-gun | 108.1 |
| Oita | 90.6 | Beppu City | 110.3 |
| Oita | 90.6 | Taketa City | 116.0 |
| Okinawa | 86.5 | Ogimi Village, Kunigami District | 106.6 |
| Okinawa | 86.5 | Nakijin Village, Kunigami District | 107.6 |
| Okinawa | 86.5 | Motobu Town, Kunigami District | 113.0 |
| Okinawa | 86.5 | Ie Village, Kunigami District | 106.6 |

***^a^*** SMR: Standardized mortality ratio

**Table G. Secondary Medical Areas with high suicide standardized mortality ratio (SMR) and municipalities with low SMR.**

| SMA ***^a^*** | SMA SMR ***^b^*** | Municipality | Municipality SMR |
| --- | --- | --- | --- |
| Nakasorachi | 121.7 | Kamisunagawa-cho, Sorachi-gun | 94.0 |
| Hidaka | 124.3 | Biratori-cho, Saru-gun | 94.0 |
| Soya | 114.5 | Esashi-cho, Esashi-gun | 94.4 |
| Soya | 114.5 | Rishiri Town, Rishiri District | 90.6 |
| Soya | 114.5 | Rishirifuji Town, Rishiri District | 94.2 |
| Hokumo | 115.9 | Kunneppu Town, Tokoro District | 88.3 |
| Kamitosan | 148.4 | Noheji-cho, Kamikita-gun | 93.8 |
| Shimokita Region | 117.8 | Oma Town, Shimokita District | 89.4 |
| Numata | 142.9 | Kawaba Village, Tone District | 93.5 |
| Tosho | 153.7 | Oshima Town | 92.2 |
| Tosho | 153.7 | Niijima Village | 90.2 |
| Fuji / Tobu | 119.9 | Nishikatsura Town, Minamitsuru District | 90.9 |
| Koban | 114.6 | Susaki City | 87.0 |
| Tagawa | 125.5 | Soeda-cho, Tagawa-gun | 90.9 |
| Kuma | 134.7 | Yunomae-cho, Kuma-gun | 93.3 |
| Izumi | 117.3 | Nagashima Town, Izumi District | 91.2 |

***^a^*** SMA: Secondary Medical Areas; ***^b^*** SMR: Standardized mortality ratio

**Table H. Secondary Medical Areas with high suicide standardized mortality ratio (SMR) and municipalities with low SMR in men.**

| SMA ***^a^*** | SMA SMR ***^b^*** | Municipality | Municipality SMR ***^b^*** |
| --- | --- | --- | --- |
| Minamioshima | 110.0 | Kikonai Town, Kamiiso District | 91.6 |
| Minamioshima | 110.0 | Nanae-cho, Kameda-gun | 88.0 |
| Nemuro | 110.8 | Betsukai-cho, Notsuke-gun | 90.6 |
| Kamitosan Region | 135.8 | Noheji-cho, Kamikita-gun | 86.9 |
| Kennan | 111.0 | Yabuki-cho, Nishishirakawa-gun | 84.4 |
| Numata | 123.0 | Kawaba Village, Tone District | 92.5 |
| Ota / Tatebayashi | 118.0 | Itakura-cho, Ora-gun | 92.7 |
| Kawagoehiki | 110.8 | Higashimatsuyama City | 84.7 |
| Kawagoehiki | 110.8 | Namegawa-cho, Hiki-gun | 89.6 |
| Kawagoehiki | 110.8 | Yoshimi-cho, Hiki-gun | 85.8 |
| Tone | 110.2 | Sugito-cho, Kitakatsushika-gun | 88.3 |
| Hokubu | 110.0 | Yorii-cho, Osato-gun | 91.8 |
| Ku Chuobu | 113.3 | Bunkyo-ku | 87.8 |
| Nishitama | 112.2 | Hinode-machi, Nishitama-gun | 90.7 |
| Kitatama Hokubu | 110.3 | Kiyose City | 87.2 |
| Kaetsu | 110.6 | Tainai City | 91.1 |
| Niikawa | 118.2 | Asahi-cho, Shimoniikawa-gun | 90.4 |
| Toyama | 118.8 | Namerikawa City | 81.0 |
| Kyoto | 111.6 | Koshu City | 91.4 |
| Fuji / Tobu | 119.5 | Uenohara City | 92.6 |
| Hokushin | 114.5 | Kijimadaira Village, Shimotakai District | 92.4 |
| Kamo | 118.2 | Minamiizu Town, Kamo District | 93.1 |
| Nagoya | 114.9 | Showa-ku, Nagoya City | 88.9 |
| Nagoya | 114.9 | Midori-ku, Nagoya City | 92.5 |
| Osaka | 124.1 | Abeno-ku, Osaka City | 91.1 |
| Kobe | 109.7 | Nishi-ku, Kobe City | 92.1 |
| Gobo | 112.3 | Mihama-cho, Hidaka-gun | 90.1 |
| Tagawa | 109.7 | Soeda-cho, Tagawa-gun | 92.7 |
| Tagawa | 109.7 | Akamura, Tagawa-gun | 92.7 |

***^a^*** SMA: Secondary Medical Areas; ***^b^*** SMR: Standardized mortality ratio

**Table I. Secondary Medical Areas with high suicide standardized mortality ratio (SMR) and municipalities with low SMR in women.**

| SMA ***^a^*** | SMA SMR ***^b^*** | Municipality | Municipality SMR |
| --- | --- | --- | --- |
| Minamioshima | 110.0 | Kikonai Town, Kamiiso District | 91.6 |
| Minamioshima | 110.0 | Nanae-cho, Kameda-gun | 88.0 |
| Nemuro | 110.8 | Betsukai-cho, Notsuke-gun | 90.6 |
| Kamitosan Region | 135.8 | Noheji-cho, Kamikita-gun | 86.9 |
| Kennan | 111.0 | Yabuki-cho, Nishishirakawa-gun | 84.4 |
| Numata | 123.0 | Kawaba Village, Tone District | 92.5 |
| Ota / Tatebayashi | 118.0 | Itakura-cho, Ora-gun | 92.7 |
| Kawagoehiki | 110.8 | Higashimatsuyama City | 84.7 |
| Kawagoehiki | 110.8 | Namegawa-cho, Hiki-gun | 89.6 |
| Kawagoehiki | 110.8 | Yoshimi-cho, Hiki-gun | 85.8 |
| Tone | 110.2 | Sugito-cho, Kitakatsushika-gun | 88.3 |
| Hokubu | 110.0 | Yorii-cho, Osato-gun | 91.8 |
| Ku-Chuo-bu | 113.3 | Bunkyo-ku | 87.8 |
| Nishitama | 112.2 | Hinode-machi, Nishitama-gun | 90.7 |
| Kitatama Hokubu | 110.3 | Kiyose City | 87.2 |
| Kaetsu | 110.6 | Tainai City | 91.1 |

***^a^*** SMA: Secondary Medical Areas; ***^b^*** SMR: Standardized mortality ratio

**Table J. Secondary Medical Areas with low suicide standardized mortality ratio (SMR) and municipalities with high SMR.**

| SMA ***^a^*** | SMA SMR ***^b^*** | Municipality | Municipality SMR ***^b^*** |
| --- | --- | --- | --- |
| Soso | 85.5 | Minamisoma City | 114.2 |
| Inba | 94.2 | Yachimata City | 116.3 |
| Inba | 94.2 | Tomisato City | 119.3 |
| Inba | 94.2 | Sakae Town, Imba District | 117.1 |
| Yokosuka / Miura | 87.2 | Miura City | 113.3 |
| Yokohama Nanbu | 86.7 | Naka-ku, Yokohama City | 129.2 |
| Josho | 94.7 | Aoki Village, Chiisagata District | 114.7 |
| Daihoku | 91.2 | Otari Village, Kitaazumi District | 113.3 |
| Nagano | 93.9 | Shinano-cho, Kamiminochi-gun | 119.6 |
| Seino | 94.7 | Yoro-cho, Yoro-gun | 114.4 |
| Hokusei | 90.5 | Inabe City | 123.0 |
| Yamashiro Kita | 84.2 | Ide-cho, Tsuzuki-gun | 124.3 |
| Fukuyama / Fuchu | 93.2 | Jinseki Kogen Town, Jinseki District | 114.8 |
| Uwajima | 95.0 | Matsuno Town, Kitauwa District | 116.0 |
| Kasuya | 93.7 | Sue-cho, Kasuya-gun | 119.4 |

***^a^*** SMA: Secondary Medical Areas; ***^b^*** SMR: Standardized mortality ratio

**Table K. Secondary Medical Areas with low suicide standardized mortality ratio (SMR) and municipalities with high SMR in men.**

| SMA ***^a^*** | SMA SMR ***^b^*** | Municipality | Municipality SMR ***^b^*** |
| --- | --- | --- | --- |
| Inba | 92.8 | Yachimata City | 119.5 |
| Inba | 92.8 | Tomisato City | 118.4 |
| Kitatama Hokubu | 89.8 | Higashimurayama City | 121.2 |
| Kawasaki Nanbu | 95.2 | Kawasaki Ward, Kawasaki City | 125.5 |
| Yokosuka / Miura | 87.2 | Miura City | 118.1 |
| Yokohama Nanbu | 83.1 | Naka-ku, Yokohama City | 120.8 |
| Josho | 95.1 | Aoki Village, Chiisagata District | 118.0 |
| Nagano | 91.5 | Shinano-cho, Kamiminochi-gun | 119.9 |
| Chitahanto | 95.1 | Mihama-cho, Chita-gun | 115.3 |
| Hokusei | 93.3 | Inabe City | 129.6 |
| Yamashiro Kita | 83.8 | Ide-cho, Tsuzuki-gun | 143.7 |
| Hiroshima | 95.1 | Naka-ku, Hiroshima City | 133.5 |
| Hiroshima | 95.1 | Akitakata City | 126.2 |
| Hiroshima | 95.1 | Akiota Town, Yamagata District | 122.5 |
| Hiroshima | 95.1 | Kitahiroshima Town, Yamagata District | 115.9 |
| Kasuya | 94.5 | Sue-cho, Kasuya-gun | 120.3 |

***^a^*** SMA: Secondary Medical Areas; ***^b^*** SMR: Standardized mortality ratio

**Table L. Secondary Medical Areas with low suicide standardized mortality ratio (SMR) and municipalities with high SMR in women.**

| SMA ***^a^*** | SMA SMR ***^b^*** | Municipality | Municipality SMR ***^b^*** |
| --- | --- | --- | --- |
| Shiribeshi | 83.1 | Iwanai Town, Iwanai District | 111.0 |
| Soso | 86.5 | Soma City | 113.6 |
| Soso | 86.5 | Minamisoma City | 108.2 |
| Hitachiota / Hitachinaka | 86.7 | Daigo Town, Kuji District | 113.4 |
| Daihoku | 86.5 | Ikeda-cho, Kitaazumi-gun | 111.4 |
| Shidahaibara | 89.7 | Makinohara City | 108.6 |
| Kaifu | 91.0 | Oharu Town, Ama District | 111.3 |
| Toyono | 87.5 | Nose Town, Toyono District | 109.3 |
| Minamikawachi | 82.8 | Osakasayama City | 119.8 |
| Towa | 86.2 | Yamazoe Village, Yamabe District | 113.6 |
| Seiwa | 88.7 | Ando Town, Ikoma District | 109.2 |
| Seiwa | 88.7 | Kammaki-cho, Kitakatsuragi-gun | 109.0 |
| Chuwa | 86.6 | Gose City | 110.4 |
| Kennan Tobu | 84.8 | Kibichuo Town, Kaga District | 109.1 |
| Tobu | 89.4 | Kamiita-cho, Itano-gun | 107.6 |
| Chubu | 86.8 | Taku City | 106.7 |
| Chubu | 86.8 | Yoshinogari Town, Kanzaki District | 111.5 |
| Nagasaki | 86.2 | Togitsu-cho, Nishisonogi-gun | 108.1 |
| Yatsushiro | 90.9 | Hikawa-cho, Yatsushiro-gun | 114.9 |
| Nobeokanishiusuki | 86.1 | Takachiho Town, Nishiusuki District | 120.3 |
| Nobeokanishiusuki | 86.1 | Hinokage Town, Nishiusuki District | 118.6 |
| Amami | 82.7 | Kikai-cho, Oshima-gun | 108.6 |
| Amami | 82.7 | China Town, Oshima District | 109.3 |

***^a^*** SMA: Secondary Medical Areas; ***^b^*** SMR: Standardized mortality ratio

**Table M. Prefectures with high suicide standardized mortality ratio (SMR) and Secondary Medical Areas with low SMR.**

| Prefecture | Prefecture SMR ***^a^*** | SMA ***^b^*** | SMA SMR ***^a^*** |
| --- | --- | --- | --- |
| Fukushima | 110.7 | Soso | 85.5 |
| Shimane | 114.7 | Oki | 92.9 |

***^a^*** SMR: Standardized mortality ratio; ***^b^*** SMA: Secondary Medical Areas

**Table N. Prefectures with high suicide standardized mortality ratio (SMR) and Secondary Medical Areas with low SMR in men.**

| Prefecture | Prefecture SMR ***^a^*** | SMA ***^b^*** | SMA SMR ***^a^*** |
| --- | --- | --- | --- |
| Saga | 112.5 | Tobu | 95.2 |
| Kagoshima | 116.4 | Kagoshima | 93.4 |

***^a^*** SMR: Standardized mortality ratio; ***^b^*** SMA: Secondary Medical Areas

**Table O. Prefectures with high suicide standardized mortality ratio (SMR) and Secondary Medical Areas with low SMR in women.**

| Prefecture | Prefecture SMR ***^a^*** | SMA ***^b^*** | SMA SMR ***^a^*** |
| --- | --- | --- | --- |
| Fukushima | 105.9 | Soso | 86.5 |
| Tochigi | 109.5 | Kensei | 87.5 |

***^a^*** SMR: Standardized mortality ratio; ***^b^*** SMA: Secondary Medical Areas

**Table P. Prefectures with low suicide standardized mortality ratio (SMR) and Secondary Medical Areas with high SMR.**

| Prefecture | Prefecture SMR ***^a^*** | SMA ***^b^*** | SMA SMR ***^a^*** | Prefecture |
| --- | --- | --- | --- | --- |
| Chiba | 97.5 | 1209 | Ichihara | 118.4 |
| Tokushima | 90.2 | 3605 | Seibu | 118.5 |

***^a^*** SMR: Standardized mortality ratio; ***^b^*** SMA: Secondary Medical Areas

**Table Q. Prefectures with low suicide standardized mortality ratio (SMR) and Secondary Medical Areas with high SMR in men.**

| Prefecture | Prefecture SMR ***^a^*** | SMA ***^b^*** | SMA SMR ***^a^*** |
| --- | --- | --- | --- |
| Chiba | 94.7 | Ichihara | 119.6 |
| Tokyo | 94.2 | Tosho | 165.2 |
| Okayama | 92.4 | Maniwa | 122.7 |
| Tokushima | 90.6 | Seibu | 120.4 |

***^a^*** SMR: Standardized mortality ratio; ***^b^*** SMA: Secondary Medical Areas
